# Statistics of Mortality from Typhoid Fever

**Published:** 1874-11-15

**Authors:** 


Statistics of Mortality from Typhoid Fever.—In answer to the inquiries of a correspondent, we state, that according to the census tables of mortality for the year 1870, the whole number of deaths from typhoid, or enteric fever, in the United States, during the year ending June 1st, 1870, was 22,187. Of these, 11,430 were males, and 10,757 were females.

During the same year the tables show the aggregate number of deaths from *typhus fever* to have been 1,770; of whom 1,004 were males and 766 females. In the State of Illinois, for the same year, the whole number of deaths reported from typhoid, or enteric fever, was 1758; of these 961 were males and 797 females. The whole number of deaths from typhus was 131; of whom 71 were males and 60 females.

